# Acupuncture Methods for Primary Trigeminal Neuralgia: A Systematic Review and Network Meta-Analysis of Randomized Controlled Trials

**DOI:** 10.1155/2022/3178154

**Published:** 2022-02-21

**Authors:** Zihan Yin, Fumin Wang, Mingsheng Sun, Ling Zhao, Fanrong Liang

**Affiliations:** School of Acu-Mox and Tuina, Chengdu University of Traditional Chinese Medicine, Chengdu, China

## Abstract

**Background:**

Primary trigeminal neuralgia (PTN) is a clinical refractory disorder characterized by excruciating pain that severely impacts the quality of life. Several studies have shown that acupuncture can improve PTN pain. However, the comparative efficacy and safety of acupuncture are unknown. Herein, a systematic review was conducted to compare the efficacy and safety of various acupuncture methods for PTN treatment.

**Methods:**

Relevant randomized controlled trials (RCTs) published up to 1 August 2021 were obtained from PubMed, Embase, Cochrane Central Register of Controlled Trials, Web of Science Core Collection, Chinese National Knowledge Infrastructure, Chinese Biomedical Literature Database, CQVIP Database, Wanfang Database, Allied and Alternative Medicine Database, and related registration platforms. Two authors independently selected the studies and obtained data. Cochrane Handbook was used to assess the methodological quality. We put the pain relief as the primary outcome and the response rate and adverse events as the secondary outcomes. Review Manager v5.3, ADDIS v1.16.8, and STATA v15.0 software were used for data analysis. The intraclass correlation coefficient was used to assess the consistency of the two investigators.

**Results:**

A total of 58 RCTs with 4,126 participants were obtained. The meta-analysis indicated that five acupuncture methods were superior to conventional medicine (carbamazepine) in pain reduction intensity and response rate. Meanwhile, electronic acupuncture plus manual acupuncture was the most effective therapy since it reduced pain intensity in 11 methods and improved the response rate in 10 interventions. Moreover, six interventions had acceptable adverse events, and none of the included studies reported severe adverse events. However, most pieces of evidence were ranked as critically low.

**Conclusion:**

These findings show that acupuncture methods can be effective and safe for PTN. Moreover, electronic acupuncture plus manual acupuncture maybe the best acupuncture treatment for PTN and should be administered to PTN patients. However, additional well-designed and high-quality RCTs should be conducted to verify the above findings in the future. The systematic review is registered with CRD42020221456.

## 1. Introduction

Primary trigeminal neuralgia (PTN) is a common neuralgia caused by the compression of an aberrant tissue associated with the trigeminal nerve [1, 2]. The latest classification system identifies TN as either classical or idiopathic TN based on the degree of neurovascular contact or secondary TN caused by pathology other than neurovascular contact [1]. Meanwhile, it is severe, unilateral, paroxysmal, and recurring pain [3] which can severely impact the quality of life [4]. Al-Quliti and colleagues illustrated that 26.8 people per 100,000 suffer from PTN, usually occurring in middle-aged and elderly people [5–7]. PTN is a global public health issue [8]. Several interventions have been used to control PTN, and carbamazepine is the common treatment method since the 1960s [9]. However, many patients do not tolerate pharmacological therapies over a long period due to the carbamazepine side effects [8, 10], thus seeking nonpharmacological treatments.

As an ancient nonpharmacological therapy, acupuncture has been used for PTN treatment in China [3, 11–15] for a long period [16]. Numerous studies have also shown that acupuncture can be used for PTN treatment [3, 11–17]. Furthermore, the acupuncture analgesic effect is associated with substance P (SP) and *β*-endorphin. Several trials have demonstrated that acupuncture can decrease SP content and enhance the expression of *β*-endorphin [18–21]. Although previous systematic reviews [3] have shown that acupuncture methods have good efficacy and safety in improving PTN symptoms, they only focused on a unitary acupuncture method against antiepileptic drugs (carbamazepine)/sham acupuncture (SA). Meanwhile, there are various acupuncture therapies. For instance, the PTN acupuncture methods include manual acupuncture (MA) therapy, acupuncture-moxibustion (AM) therapy, electronic acupuncture (EA) therapy, and fire acupuncture (FA) therapy. Therefore, it is necessary to determine the most appropriate acupuncture methods for PTN therapy.

Network meta-analysis (NMA) analyzes diversified methods from different trials and calculates their relative effects [22–24]. It was applied to compare various interventions simultaneously in a unitary analysis by combining indirect and direct evidence in a network of trials. It may also assist to determine suitable therapeutic methods and illustrate their efficacy and safe application in clinical operations, thus guiding health policies [25]. Thus, this study aimed to compare and rank the efficacy and safety of all known acupuncture therapies on PTN via NMA.

## 2. Methods

The study was registered on PROSPERO (registration number: CRD42020221456) and was conducted following the Preferred Reporting Items for Systematic Review and Meta-Analysis-Network Meta-Analysis (PRISMA-NMA) [26] and the accompanying checklist (Appendix 1).

### 2.1. Eligibility Criteria and Exclusion Criteria

The PICOS (participant, intervention, control, outcome, and study design) criteria were used for inclusion and exclusion analysis.

#### 2.1.1. Types of Studies

All RCT studies reported in English/Chinese were included, while quasi and cluster RCTs, non-RCTs, case reports, and studies with no detailed data were excluded.

#### 2.1.2. Types of Participants

All PTN patients, regardless of gender, race, and age, were included. Similarly, all types of PTN, including classical TN and idiopathic TN [1] regardless of their etiology, severity, or specific criteria diagnoses, such as diagnostic criteria or references, were included. TN patients without an association with the distinct primary disease were excluded.

#### 2.1.3. Types of Interventions

Herein, only trials using acupuncture as monotherapy or alternative treatments were included. According to Revised Standards for Reporting Interventions in Clinical Trials of Acupuncture (STRICTA) [27], the expected acupuncture methods included MA, EA, AM, FA, and the like. Moreover, articles describing combinations of these acupuncture methods with conventional medicine (CM) were included. The studies without a clear description of the acupuncture process, such as disinfection and sterilization, acupuncture manipulation, and posttreatment processes, were excluded. Besides, studies using acupoint embedding, acupoint application, acupoint injection, bee venom acupuncture, and other therapies were excluded because of using related drugs. Trials combining acupuncture and herbal medicine, cupping, and blood-letting puncture therapies were also excluded.

#### 2.1.4. Type of Controls

The comparison groups, such as the placebo group (SA), and conventional medicine (carbamazepine, the dosage of carbamazepine should be well documented) were based on different acupuncture methods.

#### 2.1.5. Types of Outcomes

The following studies were included: Those that compared and ranked the efficacy and safety of all acupuncture methods used in PTN. Pain intensity reduction determined using the Visual Analogue Scale (VAS) and Numerical Rating Scale (NRS) was the acceptable primary outcome. The VAS and NRS were converted to the 11-point NRS (0 indicates no pain, and 10 shows the most severe pain) due to their similarity [28]. The response rate and adverse events (AEs) were the secondary outcomes. Reduction of pain intensity and response rate were used to evaluate the efficacy of intervention methods, while AE was used to assess the safety of intervention methods. Outcome measures that were not relevant to PTN were excluded.

### 2.2. Search Strategy

Relevant studies were obtained from Embase, PubMed, Cochrane Central Register of Controlled Trials (CENTRAL), Web of Science Core Collection (WOS), and the Chinese database of the Chinese Biomedical Literature Database (CBM), Chinese National Knowledge Infrastructure (CNKI), Chinese Science and Technology Periodical Database (CQVIP), and Wanfang Database (WF) up to 1 August 2021. Furthermore, clinical trial registries (World Health Organization International Clinical Trials Registry Platform (WHO ICTRP), Clinical Trials, and Chinese Clinical Trial Register (ChiCTR)) and Allied and Complementary Medicine Database (AMED) were used as supplements. There were three major search terms: (1) clinical conditions: PTN, classical TN, and idiopathic TN; (2) acupuncture methods: manual acupuncture, electroacupuncture, and acupuncture-moxibustion; and (3) study design: randomized clinical controlled trial. A combination of subject terms and free words was used with “and,” “or” to connect the words. The Chinese and English search strategies were similar. The search strategies of each database are shown in Appendix 2.

### 2.3. Study Selection and Data Extraction

Two investigators independently selected the studies and extracted data. We first read the study title and abstract to identify duplicate research and then uploaded the remaining part to NoteExpress V.3.0. The two reviewers (ZY and FW) preliminary screened the titles/abstracts to find suitable articles and then downloaded and read the studies. Finally, the 2 researchers each read the residual full-text studies to select those that meet the inclusion criteria. A 3rd party (LZ or FL) resolved any controversy.

A standardized Microsoft Excel 2010 sheet was used to extract data, such as study identity (first author, publication year, and country), study design, diagnostic criteria, characteristics of patients (age, gender, and sample size), details of intervention group and control group, treatment courses, outcomes (primary and secondary outcomes), and results. The corresponding/first author of the article was requested for additional information in ambiguous or insufficient detail cases. Besides, a description was added to the final result when the data details were not available. The selection procedure was outlined following the PRISMA flowchart.

### 2.4. Study Quality Assessment

Two authors assessed the risk of bias (ROB) in all eligibility trials using the Cochrane Collaboration ROB assessment tool 2.0 [29]. Risk levels of randomization process, deviations from intended interventions, missing outcomes data, measurement of the outcome, and selection of the reported results were indicated as “low,” “some concerns,” or “high.” A 3rd party (LZ or FL) resolved any misunderstanding.

### 2.5. Statistical Analysis

RevMan 5.3 software was used for data analysis. The 3-arm trials were divided into 2-arm trials for all possible combinations. A pooled mean difference (MD) for continuous outcomes or relative risk (RR) of dichotomous variable data with 95% confidence intervals (CI) was presented for each intervention. The random effects model adopted by the DerSimonian–Laired method was used as a conservative estimate [30]. The *I*^2^ statistic and *p* values were determined for statistical heterogeneity analysis. *p* < 0.05 and *I*^2^>75% indicated significant heterogeneity. The Bayesian network analysis framework and Markov Chain Monte Carlo (MCMC) method [31] were used to assess and process a priori data via the Aggregate Data Drug Information System (ADDIS V.1.16.8 software, Drugis, Groningen, NL). The parameters were 4 Markov chains for simulation at 50,000 simulation iterations to determine their posterior distributions. Moreover, the first 20,000 simulation iterations were used to eliminate the initial value's impacts and the last 30,000 were used for sampling. The node-splitting method was used to integrate direct and indirect multiple-treatment comparisons of the RCTs [32]. STATA version 15.0 software (StataCorp LP, Texas, USA) was used for network plot analysis, where node sizes indicated the number of study patients and connection sizes showed the number of trials in each treatment. Finally, the node-splitting method was also used to assess the local inconsistency [33]. *p* < 0.05 was considered a significant statistical difference between indirect and direct multiple-treatment comparisons. Either the inconsistency model or consistency model was used. The model convergence is the potential scale reduced factor (PSRF), and PSRF close to 1 indicates successful convergence [34].

### 2.6. Publication Bias

A funnel plot was used to assess reporting bias since over 10 studies were evaluated in the meta-analysis.

### 2.7. Quality of Evidence

The Grades of Recommendations, Assessment, Development, and Evaluation (GRADE) was used to assess the overall quality of the evidence [35, 36] and ranked it as “high,” “moderate,” “low,” and “critically low.”

### 2.8. Assessing Reviewer Agreement

Since the two reviewers independently selected and extracted data and evaluated the quality of RCTs, the intraclass correlation coefficient (ICC) [37] was determined to assess their consistency (ICC score, 0.95).

## 3. Results

### 3.1. Study Selection

**Figure 1:**
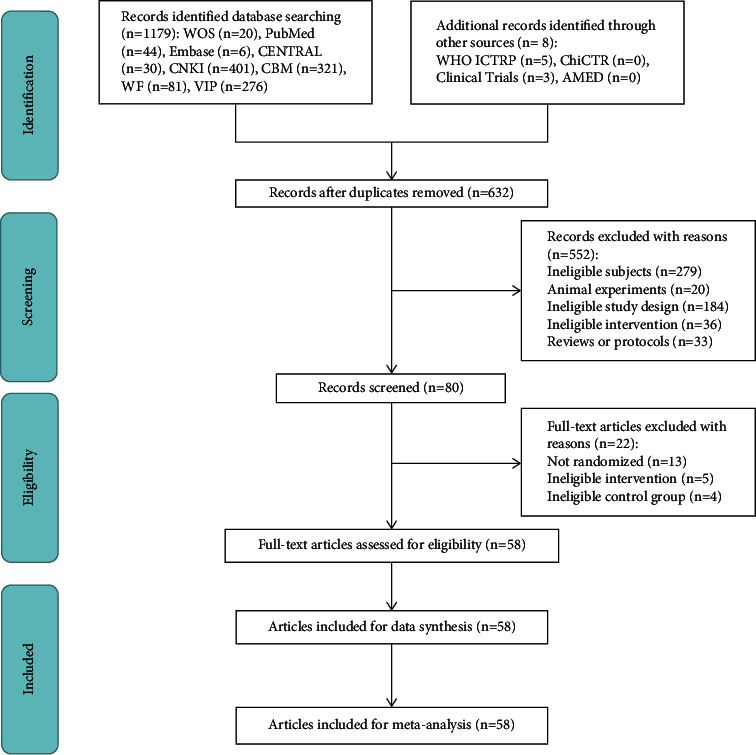
The PRISMA flowchart of selection process.

A total of 1187 studies were obtained, and only 58 RCTs [11, 17, 38–93] with 4126 patients were selected for the systematic review. The selection process is presented in Figure 1.

### 3.2. Study Characteristics

All trials were incorporated in the final Bayesian network meta-analysis. A total of 56 RCTs [11, 38–51, 53–93] were written in Chinese, while two studies [17, 52] were published in English. All the 58 articles were reported between 2004 and 2021. The studies were grouped at a 1 : 1 ratio. The ages of patients were between 38 and 64 years except for six trials, which did not indicate the ages [71, 77, 79, 88–90]. The included studies had more women than men with sample sizes between 24 and 217. The treatment duration ranged between 10 days and 90 days (mean, 20–30 days). MA, EA, AM, FA, SA, CM (carbamazepine) treatments, and their combination therapies were used in the included studies. The studies had different acupoints in all acupuncture therapies. However, the Xia Guan (ST 7), He Gu (LI4), and Tai Yang (EX-HN5) were the common acupoints. Moreover, the mean daily usage of carbamazepine was between 0.3 and 0.8 g. The response rate was the frequently used outcome measures in most studies. The characteristics of the included studies are shown in Table 1.

### 3.3. Study Quality Assessment

**Figure 2:**
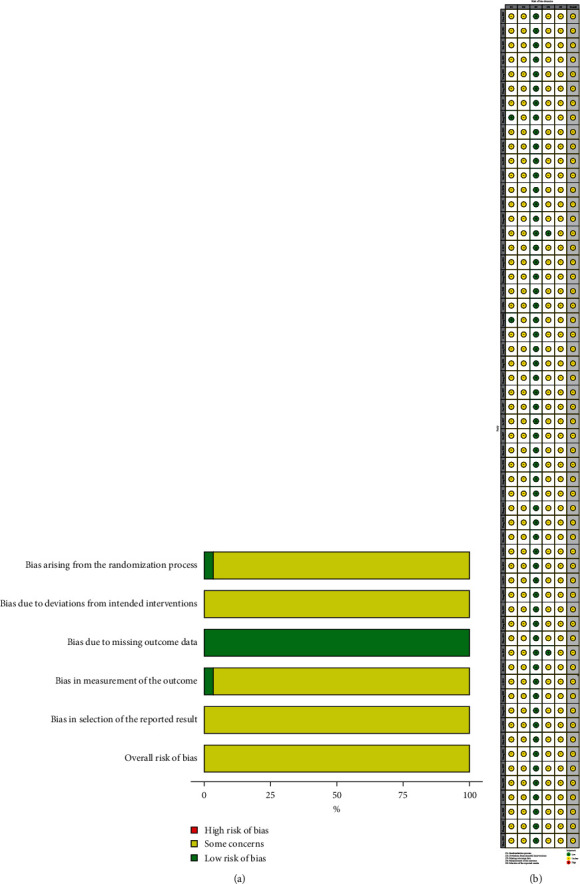
(a) Risk of bias graph; (b) risk of bias summary.

The methodology quality of the included RCTs was assessed. (1) Randomisation process: only two trials [45, 58] were ranked as “low risk” because they had detailed information, while 56 trials were ranked as “unclear” due to insufficient information. (2) Deviations from intended interventions: all trials had insufficient information on intended interventions and were ranked as “some concerns.” (3) Missing outcomes data: all RCTs were ranked as “low risk” due to the complete implementation plan. (4) Measurement of the outcome: a total of 56 trials were ranked as “some concerns” and only two [17, 73] as “low risk.” (5) Selection of the reported results: all the RCTs were ranked as “some concerns” due to insufficient information. The quality assessment detail of the RCTs is shown in Figure 2.

### 3.4. Pairwise Meta-Analysis Results

#### 3.4.1. Reduction in Pain Intensity

A total of 14 pairwise meta-analyses were conducted to compare the pain reduction intensity of various therapies (Table 2). MA was highly effective in reducing pain intensity compared to SA (1 RCT, MD, 1.66; 95% CI: 0.93–2.39) and CM (9 RCTs, MD, 1.14; 95% CI: 0.48–1.80). EA efficacy was statistically different than CM efficacy (1 RCT, MD, 1.07; 95% CI: 0.09–2.05). Furthermore, MA + EA efficacy was statistically different than MA efficacy (1 RCT, MD, 3.18; 95% CI: 2.43–3.93) and EA efficacy (2 RCTs, MD, 1.25; 95% CI: 0.78–1.72). MA + CM (7 RCTs, MD, 1.19; 95% CI: 0.55–1.84), AM + CM (3 RCTs, MD, 1.88; 95% CI: 0.87–2.90), and EA + CM (2 RCTs, MD, 1.22; 95% CI: 0.42–2.02) were highly effective in reducing pain intensity than CM. However, MA + CM efficacy was statistically different than SA + CM efficacy (1 RCT, MD, 1.60; 95% CI: 0.32–2.88). Besides, no statistical difference was found between MA and AM, MA and FA, EA and MA, EA and FA, AM and CM, EA + CM, and MA + CM.

#### 3.4.2. Response Rate

A total of 13 pairwise meta-analyses were conducted to compare the response rates of different treatments (Table 3). MA efficacy was significantly different than the CM efficacy (25 RCTs, RR, 1.21; 95% CI: 1.15–1.27) and SA efficacy (1 RCT, RR, 1.68; 95% CI: 1.17–2.42). AM + CM (3 RCTs, RR, 1.25; 95% CI: 1.10–1.42) and MA + CM (10 RCTs, RR, 1.20; 95% CI: 1.14–1.26) were highly effective compared with CM. MA + EA was highly effective than MA (1 RCT, RR, 1.50; 95% CI: 1.13–1.99) and EA (2 RCTs, RR, 1.33; 95% CI: 1.03–1.72). However, no statistical difference was found between MA and FA, MA and AM, EA and MA, EA/AM and CM, EA + CM, and CM/MA + CM.

### 3.5. Network Meta-Analysis Results

#### 3.5.1. Network Plot of Different Interventions

**Figure 3:**
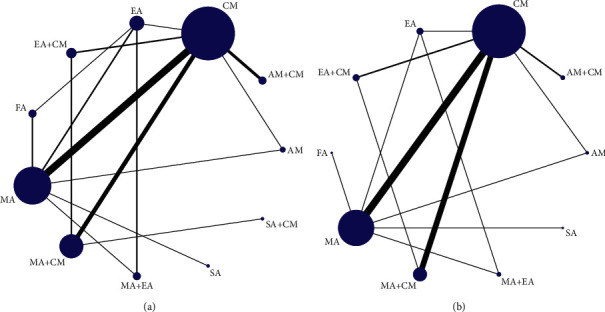
The network graph of different interventions of (a) pain relief and (b) response rate.

A total of 11 therapies were used (AM, AM + CM, EA, EA + CM, FA, MA, MA + CM, MA + EA, CM, SA, and SA + CM). The network plots of these treatments are shown in Figure 3. A total of 36 trials with 2,308 participants using 11 methods indicated pain intensity reduction (Figure 3(a)), while 53 RCTs with 3,744 participants using 10 therapies showed a response rate (Figure 3(b)).

#### 3.5.2. Statistical Inconsistency Analysis

The node-splitting method was used for local inconsistency analysis. The direct and indirect effects had no statistically different reduction of pain intensity and response rate (*p* ≥ 0.05).

#### 3.5.3. Reduction in Pain Intensity

**Figure 4:**
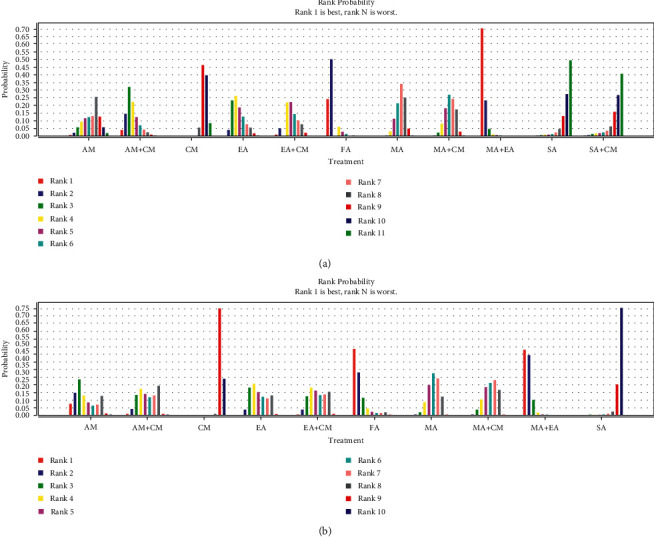
The figure of ranking probability of (a) pain relief and (b) response rate.

STATA 15.0 was used for network plot analysis of the 11 treatments (Figure 3(a). The Bayesian network meta-analysis was conducted using the consistency model to produce the ranking probability plot since the PSRF scores were close to 1 (Appendix 3(a)) and *p* values were higher than 0.05 (Appendix 3(b)). MA + EA, FA, and AM + CM significantly reduced pain intensity (Figure 4(a)). However, MA + EA was the optimum intervention method. The meta-analyses details are shown in Table 4. MA + EA was more effective than EA, MA, MA + CM, AM, CM, SA, and SA + CM. Furthermore, MA + EA and FA were more effective than MA, SA + CM, SA, and CM. MA + EA, FA, AM + CM, EA, EA + CM, MA, and MA + CM were more effective than CM. AM + CM was significantly effective than SA.

#### 3.5.4. Response Rate

STATA 15.0 was also used for network plot analysis of 10 interventions (Figure 3(b)). Similarly, the Bayesian meta-analysis was conducted using the consistency model to produce the ranking probability plot (Figure 4(b)) since the PSRF scores were close to 1 (Appendix 4(a)) and *p* values were higher than 0.05 (Appendix 4(b)). MA + EA, FA, and AM had the most significant response rates (Figure 4(b)). However, MA + EA/FA maybe the optimum intervention method. The meta-analyses details are shown in Table 5. MA + EA was more effective than 4 interventions (EA, MA, CM, and SA). MA + EA, MA + CM, MA, EA + CM, and AM + CM were more effective than CM, and MA + EA, FA, AM, EA, MA, EA + CM, and AM + CM were highly effective than SA.

### 3.6. Safety

A total of 24 studies using MA, CM, SA, EA + CM, AM + CM, and MA + CM demonstrated adverse effects, with 14 studies indicating 25 MA-related adverse events. Besides, 22 studies described 205 CM-related adverse events and one study showed one SA-related adverse event. Three studies reported 52 MA + CM-related adverse events, one study showed seven EA + CM-related adverse events, and one study reported two AM + CM-related adverse events. The details of adverse events between acupuncture and the CM group are shown in Table 6.

### 3.7. Heterogeneity

RevMan 5.3 was used for sensitivity analysis to evaluate the stability and reliability of the results. The results were shown to be considerably reliable and stable and could have high clinical heterogeneity due to the diverse selection of acupoints, duration of treatment, carbamazepine dose, and the like.

### 3.8. Publication Bias

**Figure 5:**
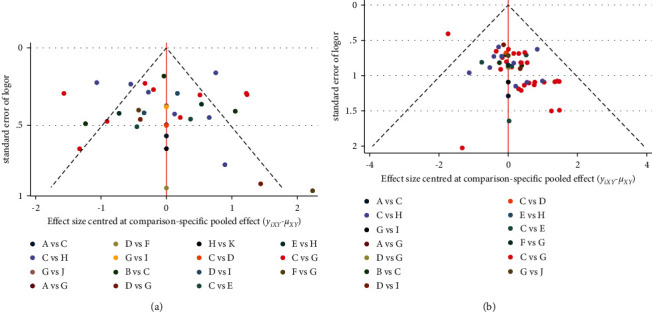
(a) Funnel plot for the network meta-analysis of pain relief. (b) Funnel plot for the network meta-analysis of response rate. Note. A: AM; B: AM+ CM; C: CM; D: EA; E: EA + CM; F: FA; G: MA; H: MA+ CM; I: MA+ EA; J: SA; K: SA +CM.

A funnel graph was used to determine the reporting bias assessment (Figure 5). The graph indicated a low risk of publication bias, as shown in the comparison-adjusted funnel plots for reducing pain intensity (Figure 5(a)). However, the funnel plots of response rate indicated a potential reporting bias (Figure 5(b)).

### 3.9. Quality of Evidence

The GRADE criteria were used to compare the direct and indirect evidence. The quality of outcomes was critically low to moderate. However, most evidence were critically low (Appendixes 5 and 6), mainly due to the ROB, inconsistency, and imprecision.

## 4. Discussion

Although CM (carbamazepine) is used for PTN treatment, it has some inevitable adverse effects [94]. In this study, alternative PTN treatments were explored. Acupuncture has been successfully adopted for PTN treatment for a long time. However, several acupuncture therapies used are not regulated and standardized. NMA is used when there is no possibility of head-to-head or direct comparison of the intervention of interest versus control. Thus, it is applied to analyze several RCTs with various acupuncture therapies and rank these interventions [95]. This is the first study to conduct a Bayesian network meta-analysis to assess the efficacy of different acupuncture methods for PTN treatment based on the PRISMA-NMA.

### 4.1. Summary of Evidence

In this study, the efficacy of various therapies for PTN treatment was compared and reliable results were obtained [96, 97]. For reduction of pain intensity, EA + MA efficacy was statistically different compared with EA and MA efficacies. MA/EA efficacy was also statistically significant compared with CM efficacy. Furthermore, MA/EA/AM combined with CM showed statistically significant efficacy compared with CM. FA and SA also had different efficacies. EA + MA was the optimal acupuncture method for pain intensity reduction. EA + MA significantly reduced pain intensity compared with EA, MA, MA + CM, AM, CM, SA, and SA + CM. MA + EA, FA, AM + CM, EA, EA + CM, MA, and MA + CM were more effective than CM. For the response rate, MA and CM had different efficacies. MA + EA, MA + CM, MA, EA + CM, and AM + CM were more effective than CM. MA + EA, FA, AM, EA, MA, EA + CM, and AM + CM were highly effective than SA. EA + MA/FA was the optimal acupuncture treatment for improved response rate. However, most evidence had a critically low quality. In safety, 24 RCTs (41.38%) showed adverse events and 22 RCTs (37.93%) reported carbamazepine-related adverse events (dizziness, drowsiness, and gastrointestinal reaction). However, 15 RCTs (25.86%) reported acceptable acupuncture-related adverse events (dizziness, drowsiness, and fatigue). No study reported severe adverse events.

### 4.2. Strengths and Limitations

This study has several strengths. (a) This is the first network meta-analysis to include 58 RCTs and compare different acupuncture methods, such as AM, EA, FA, MA, and their combinations with CM, or acupuncture versus sham acupuncture and CM groups for PTN treatment; (b) this systematic review was registered on PROSPERO and followed the PRISMA-NMA guidelines to reduce ROB; (c) ICC was used to assess the reviewers' agreements to ensure the reliability of the assessment results; (d) GRADE was used to assess the quality of evidence.

However, this study has some limitations. First, the evidences were of low quality, and several limitations, including study design, implementation, analysis, and publication, influence ROB. Second, while there were strict criteria to control the quality of evidences, the included studies had several therapies and can affect the evidence quality. Third, the results had a statistically significant heterogeneity possibly due to age, gender, treatments, details of acupuncture, carbamazepine dose, or other factors from the PTN patients. Besides, most included RCTs had no follow-up time and the long-term effect of acupuncture could not be determined. Finally, all the included studies were from China and the search strategies were limited to English and Chinese, thus regional limitation.

### 4.3. Suggestions to Future Research

In this study, the methodological quality of all trials was moderate, but the quality of evidences was critically low, possibly due to the exclusion of several details (randomization, deviations from intended interventions, outcome measurement, and selective bias). Therefore, the project design, implementation, analysis, and writing of studies should strictly follow the latest edition of Cochrane Handbook for Systematic Reviews [98], the Consolidated Standards of Reporting Trials (CONSORT) [99], and STRICTA [27] in the future. Second, this study did not show the long-term efficacy and safety of acupuncture methods. Therefore, it is important to consider longitudinal trials to explore long-term efficacy and safety. Moreover, several factors, such as acupoint selection, treatment duration, intervention time, and dose of carbamazepine, impact heterogeneity. Therefore, future studies should standardize and regulate the acupuncture and CM details for PTN treatment. Finally, other core outcomes, such as changes in the psychological situation and quality of life, should be considered [100].

## 5. Conclusion

In conclusion, acupuncture reduces pain intensity, improves response rate, and has less adverse events on PTN patients. Moreover, five acupuncture therapies are superior to conventional medicine (carbamazepine). In this study, EA + MA was regarded as the optimal therapy for PTN. However, the overall quality of evidences from included studies was critically low. Therefore, well-designed and high-quality clinical trials are needed to confirm the abovementioned findings in the future.

## Figures and Tables

**Table 1:** Main characteristics of included RCTs.

Study	Country	Sample size	Allocation ratio	Age	Gender (M : F)	A	B	C	Main acupuncture points used	Duration of treatment	Efficacy and safety criteria	Main results
						Treatment group	Control group I	Control group II				
Chen (2021) [38]	China	80	1 : 1	A: 43.28 ± 6.43 B: 43.75 ± 5.89	A: (16 : 24) B: (18 : 22)	MA	CM (0.4–0.8 g/day dose of carbamazepine)	—	Tai Yang (EX-HN 5), Feng Chi (GB 20), He Gu (LI 4), and Shui Gou (DU 26)	28 days	1. Reduction of pain intensity 2. Response rate 3. Adverse events	1. A > B 2. A > B 3. A > B
Chi (2021) [39]	China	78	1 : 1	A: 53.87 ± 6.13 B: 53.18 ± 6.69	A: (21 : 18) B: (20 : 19)	MA + CM	CM (0.3 g/day dose of carbamazepine)	—	Feng Chi (GB 20), Tian Zhu (BL 10), Wai Guan (SJ 5), Wan Gu (GB 12), He Gu (LI 4), Yin Tang (DU 29), and Shen Ting (DU 24)	30 days	1. Response rate 2. Adverse events	1. A > B 2. A > B
Niu (2021) [40]	China	72	1 : 1	A: 56.37 ± 3.21 B: 56.43 ± 3.25	A: (16 : 20) B: (15 : 21)	MA + CM	CM (0.3 g/day dose of carbamazepine)	—	Wan Gu (GB 12), Yin Tang (DU 29), Shen Ting (DU 24), Feng Chi (GB 20), Wai Guan (SJ 5), He Gu (LI 4), and Tian Zhu (BL 10)	30 days	1. Reduction of pain intensity 2. Response rate	1. A > B 2. A > B
Qin (2021) [41]	China	66	1 : 1	A: 54.5 ± 7.8 B: 55.2 ± 6.3	A: (15 : 16) B: (14 : 19)	MA + CM	CM (0.3–0.6 g/day dose of carbamazepine)	—	Yu Yao (EX-HN 4), Jia Che (ST 6), Xia Guan (ST 7), Si Bai (ST 2), Ashi point, Cheng Jiang (RN 24), Nei Guan (PC 6), and He Gu (LI 4)	30 days	1. Reduction of pain intensity 2. Response rate	1. A > B 2. A > B
Wang (2021) [42]	China	72	1 : 1	42.54 ± 6.84	29 : 43	AM + CM	CM (0.4–0.8 g/day dose of carbamazepine)	—	Feng Chi (GB 20), Yin Tang (DU 29), Shen Ting (DU 24), He Gu (LI 4), Tai Chong (LR 3), Nei Ting (ST 44), and Cuan Zhu (BL 2)	30 days	1. Reduction of pain intensity 2. Response rate	1. A > B 2. A > B
Wang (2020) [43]	China	42	1 : 1	A: 46.98 ± 8.12 B: 45.61 ± 7.33	A: (9 : 12) B: (7 : 14)	MA	CM (0.5–0.8 g/day dose of carbamazepine)	—	Tai Yang (EX-HN 5), Bai Hui (DU 20), Yin Tang (DU 29), He Gu (LI 4), and Xia Guan (ST 7)	90 days	1. Response rate	1. A > B
Ta (2020) [44]	China	72	1 : 1	A: 48.64 ± 10.39 B: 46.69 ± 9.91	A: (22 : 14) B: (18 : 18)	MA + CM	CM (0.6 g/day dose of carbamazepine)	—	Xia Guan (ST 7), Quan Liao (SI 18), Ying Xiang (LI 20), Si Bai (ST 2), Di Cang (ST 4), Jia Che (ST 6), Xia He Kong, Ke Kong, Nei Ting (ST 44), and He Gu (LI 4)	30 days	1. Reduction of pain intensity	1. A > B
Zhang (2019) [45]	China	64	17 : 15	A: 47.3 ± 5.7 B: 45.8 ± 9.2	A: (12 : 22) B: (11 : 19)	MA	CM (0.3–0.6 g/day dose of carbamazepine)	—	Quan Liao (SI 18), Yang Ling Quan (GB 34), and Feng Long (ST 40)	28 days	1. Reduction of pain intensity 2. Response rate 3. Adverse events	1. A > B 2. A > B 3. A > B
Hao (2019) [46]	China	54	1 : 1	A: 45.6 ± 3.4 B: 47.2 ± 3.8	A: (12 : 15) B: (13 : 14)	MA + CM	CM (0.4–0.8 g/day dose of carbamazepine)	—	Qu Bin (GB 7), Cuan Zhu (BL 2), Si Bai (ST 2), Jia Cheng Jiang, Xia Guan (ST 7), Tai Yang (EX-HN 5), Shuai Gu (GB 8), Feng Chi (GB 20), Bai Hui (DU 20), He Gu (LI 4), Tai Chong (LR 3), and Wai Guan (SJ 5)	30 days	1. Reduction of pain intensity 2. Response rate	1. A > B 2. A > B
Wu (2019a) [47]	China	28	1 : 1	A: 45.24 ± 10.78 B: 47.06 ± 11.02	A: (5 : 9) B: (4 : 10)	AM	CM (0.4–0.8 g/day dose of carbamazepine)	—	Xia Guan (ST 7)	30 days	1. Reduction of pain intensity 2. Response rate	1. A > B 2. A < B
Xu (2019) [48]	China	66	1 : 1	A: 54.5 ± 2.2 B: 54.8 ± 1.9	A: (18 : 15) B: (19 : 14)	AM + CM	CM (0.3 g/day dose of carbamazepine)	—	Yu Yao (EX-HN 4), Ying Xiang (LI 20), Ting Gong (SI 19), Yang Bai (GB 14), Si Bai (ST 2), Jia Cheng Jiang, Xia Guan (ST 7), Di Cang (ST 4), Jia Che (ST 6), Zu San Li (ST 36), A Shi, and He Gu (LI 4)	20 days	1. Reduction of pain intensity 2. Response rate 3. Adverse events	1. A > B 2. A > B 3. A > B
Mu (2019) [49]	China	100	1 : 1	A: 42.6 ± 5.7 B: 43.1 ± 5.4	A: (23 : 27) B: (22 : 28)	MA	CM (0.2 g/day dose of carbamazepine)	—	Yin Tang (DU 29), Shui Gou (DU 26), Shen Ting (DU 24), Wan Gu (GB 12), Tian Zhu (BL 10), Feng Chi (GB 20), He Gu (LI 4), and Wai Guan (SJ 5)	30 days	1. Response rate	1. A > B
Wu (2019b) [50]	China	60	1 : 1	A: 48.2 ± 10.1 B: 48.3 ± 10.7	A: (15 : 15) B: (15 : 15)	MA	AM	—	Xia Guan (ST 7)	30 days	1. Reduction of pain intensity 2. Response rate	1. A < B 2. A < B
Liu (2019) [51]	China	88	1 : 1	A: 48.72 ± 5.27 B: 48.34 ± 5.72	A: (17 : 27) B: (19 : 25)	MA	CM (0.4–0.8 g/day dose of carbamazepine)	—	Feng Chi (GB 20), Shen Ting (DU 24), He Gu (LI 4), Yin Tang (DU 29), Wan Gu (GB 12), Tian Zhu (BL 10), and Shui Gou (DU 26)	30 days	1. Reduction of pain intensity 2. Response rate 3. Adverse events	1. A > B 2. A > B 3. A > B
Wang (2019) [52]	China	30	1 : 1	A: 45.8 ± 7.9 B: 44.8 ± 7.7	A: (7 : 8) B: (8 : 7)	MA	CM (0.5–0.8 g/day dose of carbamazepine)	—	He Gu (LI 4), Yin Tang (DU 29), Xia Guan (ST 7), Tai Yang (EX-HN 5), and Bai Hui (DU 20)	90 days	1. Response rate	1. A > B
Gao (2019) [17]	China	126	31 : 32	A: 63.97 ± 13.63 B: 63.96 ± 11.81	A: (20 : 42) B: (19 : 35)	MA + CM (0.6–1.2 g/day dose of carbamazepine)	SA + CM (0.6–1.2 g/day dose of carbamazepine)	—	MA/SA: Nei Ting (ST 44), He Gu (LI 4), San Jian (LI 3), Yu Yao (EX-HN 4), Cuan Zhu (BL 2), Yang Bai (GB 14), Quan Liao (SI 18), Si Bai (ST 2), Ju Liao (ST 3), Jia Che (ST 6), and Xia Guan (ST 7)	70 days	1. Reduction of pain intensity	1. A > B
Si (2018) [53]	China	66	1 : 1	A: 57.12 ± 7.89 B: 56.36 ± 7.56	A: (12 : 21) B: (13 : 20)	EA + CM	CM (0.3–0.6 g/day dose of carbamazepine)	—	Tai Yang (EX-HN 5), Si Bai (ST 2), Xia Guan (ST 7), Feng Chi (GB 20), Jia Che (ST 6), and Cheng Jiang (RN 24)	30 days	1. Reduction of pain intensity 2. Response rate 3. Adverse events	1. A > B 2. A > B 3. A > B
Liang (2018) [54]	China	86	1 : 1	A: 45.3 ± 4.6 B: 45.6 ± 4.3	A: (20 : 23) B: (18 : 25)	MA	CM (0.4–0.8 g/day dose of carbamazepine)	—	Feng Chi (GB 20), Shou San Li (LI 10), Xia Guan (ST 7), Yi Feng (SJ 17), He Gu (LI 4), Tou Wei (ST 8), Yang Bai (GB 14), Tai Yang (EX-HN 5), Si Bai (ST 2), Ting Hui (GB 2), Ying Xiang (LI 20), and Cheng Jiang (RN 24)	30 days	1. Response rate 2. Adverse events	1. A > B 2. A > B
Huang (2018a) [55]	China	64	1 : 1	A: 44.86 ± 6.39 B: 43.64 ± 5.47	A: (12 : 21) B: (11 : 20)	MA	CM (0.4–0.8 g/day dose of carbamazepine)	—	Tai Yang (EX-HN 5), Xia Guan (ST 7), Feng Chi (GB 20), Jia Che (ST 6), He Gu (LI 4), and Di Cang (ST 4)	30 days	1. Reduction of pain intensity 2. Response rate 3. Adverse events	1. A > B 2. A > B 3. A > B
Yan (2018) [56]	China	72	1 : 1	A: 43.13 ± 6.45 B: 44.18 ± 7.34	A: (14 : 22) B: (11 : 25)	MA	CM (0.6 g/day dose of carbamazepine)	—	Xia Guan (ST 7), Feng Chi (GB 20), Jia Che (ST 6), He Gu (LI 4), and Di Cang (ST 4)	30 days	1. Response rate 2. Adverse events	1. A > B 2. A > B
Li (2018a) [57]	China	56	1 : 1	A: 44.64 ± 9.86 B: 44.89 ± 7.71	A: (11 : 17) B: (12 : 16)	MA + EA	MA	—	MA: Xia Guan (ST 7), Si Bai (ST 2), Tai Chong (LR 3), Nei Ting (ST 44), Jia Che (ST 6), He Gu (LI 4), Di Cang (ST 4), Yang Bai (GB 14), Cuan Zhu (BL 2), Quan Liao (SI 18), Ju Liao (ST 3), Cheng Jiang (RN 24 EA: Xia Guan (ST 7), and Di Cang (ST 4))	14 days	1. Reduction of pain intensity 2. Response rate 3. Adverse events	1. A > B 2. A > B 3. A > B
Huang (2018b) [58]	China	60	1 : 1	A: 51.30 ± 13.77 B: 53.10 ± 13.83	A: (14 : 16) B: (13 : 17)	FA	MA	—	FA/MA: Xia Guan (ST 7), A Shi, He Gu (LI 4), and Tai Chong (LR 3)	24 days	1. Reduction of pain intensity 2. Response rate	1. A > B 2. A > B
Li (2018b) [59]	China	88	1 : 1	A: 42.03 ± 2.56 B: 41.20 ± 2.33	A: (21 : 23) B: (20 : 24)	MA	CM (0.4–0.8 g/day dose of carbamazepine)	—	Yin Tang (DU 29), Shen Ting (DU 24), He Gu (LI 4), Wan Gu (GB 12), Tian Zhu (BL 10), Shui Gou (DU 26), Feng Chi (GB 20), and Wai Guan (SJ 5)	30 days	1. Response rate 2. Adverse events	1. A > B 2. A > B
Long (2018) [60]	China	70	1 : 1	A: 55.23 ± 6.16 B: 55.10 ± 5.05	A: (13 : 22) B: (14 : 21)	AM + CM	CM (0.4–0.8 g/day dose of carbamazepine)	—	Xia Guan (ST 7) and A Shi	21 days	1. Reduction of pain intensity 2. Response rate	1. A > B 2. A > B
Ying (2018) [61]	China	124	1 : 1	A: 62.37 ± 5.41 B: 62.11 ± 5.28	A: (25 : 37) B: (27 : 35)	EA + CM	CM (0.3–0.4 g/day dose of carbamazepine)	—	Tai Yang (EX-HN 5), Si Bai (ST 2), Tou Wei (ST 8), Xia Guan (ST 7), Quan Liao (SI 18), Jia Che (ST 6), Cheng Jiang (RN 24), Cheng Jiang (RN 24), Feng Chi (GB 20), He Gu (LI 4), and Tai Chong (LR 3)	28 days	1. Response rate	1. A > B
Huang (2017) [11]	China	36	1 : 1	A: 49 ± 14 B: 49 ± 12	A: (8 : 10) B: (9 : 9)	MA + EA	EA	—	A Shi, Tai Chong (LR 3), and He Gu (LI 4)	20 days	1. Reduction of pain intensity 2. Response rate	1. A > B 2. A > B
Guo (2017) [62]	China	40	1 : 1	A: 53 ± 7 B: 56 ± 7	A: (16 : 4) B: (16 : 4)	EA + CM (0.3–0.4 g/day dose of carbamazepine)	MA + CM (0.3–0.4 g/day dose of carbamazepine)	—	EA: Jia Ji (EX-B2) MA: Tai Chong (LR 3), He Gu (LI 4), Xia Guan (ST 7), Si Bai (ST 2), Nei Ting (ST 44), Di Cang (ST 4), and Cuan Zhu (BL 2)	21 days	1. Reduction of pain intensity	1. A = B
Pan (2017) [63]	China	62	1 : 1	A: 54 ± 11 B: 59 ± 11	A: (13 : 18) B: (12 : 18)	MA + CM	CM (0.4–0.8 g/day dose of carbamazepine)	—	Auricular point (Xin, Fei, Shen Men), An Mian, Ying Xiang (LI 20), and Zu San Li (ST 36)	28 days	1. Reduction of pain intensity 2. Response rate	1. A = B 2. A > B
Su (2017) [64]	China	60	1 : 1	A: 47 ± 9.5 B: 48.33 ± 10.2	A: (9 : 21) B: (10 : 20)	EA	MA	—	Han Yan (GB 4), Xuan Li (GB 6), Shuai Gu (GB 8), Qu Bin (GB 7), Xia Guan (ST 7), and He Gu (LI 4)	21 days	1. Reduction of pain intensity 2. Response rate 3. Adverse events	1. A > B 2. A > B 3. A = B
He (2017) [65]	China	62	1 : 1	A: 61.51 ± 10.55 B: 57.03 ± 11.78	A: (16 : 15) B: (17 : 14)	MA + EA	EA	—	MA + EA: Quan Xi points MA: He Gu (LI 4), Xia Guan (ST 7), Tai Yang (EX-HN 5), Tai Chong (LR 3), Nei Ting (ST 44), and Jia Che (ST 6)	20 days	1. Reduction of pain intensity 2. Response rate	1. A > B 2. A > B
Shen (2016) [66]	China	80	1 : 1	A: 59.57 ± 6.27 B: 59.82 ± 6.82	A: (23 : 17) B: (22 : 18)	MA + CM	CM (0.5–0.8 g/day dose of carbamazepine)	—	Tai Yang (EX-HN 5)	30 days	1. Reduction of pain intensity 2. Response rate 3. Adverse events	1. A > B 2. A > B 3. A > B
Xiao (2016) [67]	China	100	1 : 1	A: 54.4 ± 10.8 B: 54.2 ± 11.5	A: (13 : 37) B: (15 : 35)	MA	CM (0.4 g/day dose of carbamazepine)	—	He Gu (LI 4), Xue Hai (SP 10), and Ge Shu (BL 17)	28 days	1. Reduction of pain intensity 2. Response rate 3. Adverse events	1. A > B 2. A > B 3. A > B
Feng (2016) [68]	China	217	1 : 1	A: 58.4 ± 4.3 B: 58.3 ± 4.2	A: (45 : 64) B: (46 : 62)	MA + CM	CM (0.3 g/day dose of carbamazepine)	—	Shui Gou (DU 26), Yin Tang (DU 29), Wai Guan (SJ 5), and Tian Zhu (BL 10)	28 days	1. Response rate	1. A > B
Li (2016) [69]	China	50	1 : 1	60.1 ± 3.4	31 : 19	MA	CM (0.6 g/day dose of carbamazepine)	—	Feng Chi (GB 20), He Gu (LI 4), Wai Guan (SJ 5), Yin Tang (DU 29), Shen Ting (DU 24), Shui Gou (DU 26), Wan Gu (GB 12), and Tian Zhu (BL 10)	30 days	1. Response rate 2. Adverse events	1. A > B 2. A > B
Zhang (2016) [70]	China	166	1 : 1	A: 45.3 ± 2.3 B: 45.5 ± 2.2	A: (47 : 36) B: (48 : 35)	MA + CM	CM (0.6 g/day dose of carbamazepine)	—	He Gu (LI 4), Feng Chi (GB 20), Jia Che (ST 6), Tai Chong (LR 3), Nei Ting (ST 44), Xia Guan (ST 7), and Di Cang (ST 4)	30 days	1. Response rate	1.A > B
Wang (2016) [71]	China	60	1 : 1	—	A: (14 : 16) B: (13 : 17)	EA + CM (0.2 g/day dose of carbamazepine)	MA + CM (0.2 g/day dose of carbamazepine)	—	He Gu (LI 4), Tai Chong (LR 3), Nei Ting (ST 44), Xia Guan (ST 7), Di Cang (ST 4), and Si Bai (ST 2)	14 days	1. Reduction of pain intensity 2. Response rate	1. A > B 2. A > B
Zhou (2016) [72]	China	65	1 : 1	A: 42.2 ± 6.1 B: 43.5 ± 5.8	A: (20 : 13) B: (19 : 13)	MA	CM (0.6 g/day dose of carbamazepine)	—	Feng Chi (GB 20), He Gu (LI 4), Yin Tang (DU 29), Shen Ting (DU 24), Shui Gou (DU 26), Wan Gu (GB 12), and Tian Zhu (BL 10)	30 days	1. Response rate 2. Adverse events	1. A > B 2. A > B
Liu (2016) [73]	China	60	1 : 1	A: 42.86 ± 6.28 B: 42.67 ± 5.84	A: (11 : 19) B: (10 : 20)	MA	CM (0.6 g/day dose of carbamazepine)	—	Feng Chi (GB 20), He Gu (LI 4), Wai Guan (SJ 5), Yin Tang (DU 29), Shen Ting (DU 24), Shui Gou (DU 26), Wan Gu (GB 12), and Tian Zhu (BL 10)	28 days	1. Response rate 2. Adverse events	1. A > B 2. A > B
Xie (2016) [74]	China	80	1 : 1	40.1 ± 0.2	—	MA	CM (0.6 g/day dose of carbamazepine)	—	Feng Chi (GB 20), He Gu (LI 4), Wai Guan (SJ 5), Yin Tang (DU 29), Shen Ting (DU 24), Shui Gou (DU 26), Wan Gu (GB 12), and Tian Zhu (BL 10)	30 days	1. Response rate 2. Adverse events	1. A > B 2. A > B
Liu (2015) [75]	China	84	1 : 1	A: 54.27 ± 3.15 B: 53.71 ± 3.40	A: (20 : 22) B: (17 : 25)	MA	CM (0.6 g/day dose of carbamazepine)	—	Feng Chi (GB 20), He Gu (LI 4), Wai Guan (SJ 5), Yin Tang (DU 29), Shen Ting (DU 24), Shui Gou (DU 26), Wan Gu (GB 12), and Tian Zhu (BL 10)	28 days	1. Reduction of pain intensity 2. Response rate 3. Adverse events	1. A > B 2. A > B 3. A > B
Wang (2015) [76]	China	70	1 : 1	51.3 ± 8.9	30 : 40	MA	CM (0.45–63 g/day dose of carbamazepine)	—	Feng Chi (GB 20), He Gu (LI 4), Wai Guan (SJ 5), Yin Tang (DU 29), Shen Ting (DU 24), Shui Gou (DU 26), Wan Gu (GB 12), and Tian Zhu (BL 10)	30 days	1. Response rate 2. Adverse events	1. A > B 2. A > B
Xia (2015) [77]	China	60	1 : 1	—	A: (17 : 13) B: (14 : 16)	MA	CM (0.6 g/day dose of carbamazepine)	—	Feng Chi (GB 20), He Gu (LI 4), Wai Guan (SJ 5), Yin Tang (DU 29), Shen Ting (DU 24), Shui Gou (DU 26), Wan Gu (GB 12), and Tian Zhu (BL 10)	30 days	1. Response rate 2. Adverse events	1. A > B 2. A > B
Wang (2014) [78]	China	38	1 : 1	59.3 ± 2.5	23 : 15	MA	CM (0.6 g/day dose of carbamazepine)	—	Feng Chi (GB 20), He Gu (LI 4), Wai Guan (SJ 5), Shen Ting (DU 24), Shui Gou (DU 26), and Tian Zhu (BL 10)	30 days	1. Response rate	1. A > B
Zhou (2014) [79]	China	60	1 : 1	—	25 : 35	EA	CM (0.2 g/day dose of carbamazepine)	—	He Gu (LI 4) and Xia Guan (ST 7)	28 days	1. Reduction of pain intensity 2. Response rate	1. A > B 2. A > B
Liu (2014) [80]	China	60	1 : 1	A: 50.20 ± 8.47 B: 48.37 ± 7.97	A: (9 : 21) B: (11 : 17)	MA	SA	—	MA: He Gu (LI 4), Xia Guan (ST 7), Di Cang (ST 4), and Si Bai (ST 2) SA: 1 cm lateral to the MA points	20 days	1. Reduction of pain intensity 2. Response rate 3. Adverse events	1. A > B 2. A > B 3. A > B
Xie (2014) [81]	China	63	20 : 21 : 22	A: 55.2 ± 6.9 B: 55.1 ± 6.8 C: 55.2 ± 6.8	A: (5 : 15) B: (5 : 16) C: (5 : 17)	EA	MA	FA	MA: Si Bai (ST 2), Jia Che (ST 6), Di Cang (ST 4), Yang Bai (GB 14), Quan Liao (SI 18), Cheng Jiang (RN 24), Ying Xiang (LI 20), Shui Gou (DU 26), Tai Yang (EX-HN 5), and Yu Yao (EX-HN 4) EA: A Shi and the same acupoints as MA FA: Xia Guan (ST 7) and the rest are the same as MA	30 days	1. Reduction of pain intensity	1. A > B
Li (2014) [82]	China	60	1 : 1	52.0 ± 3.5	19 : 41	EA + CM	CM (0.2–0.4 g/day dose of carbamazepine)	—	Si Bai (ST 2), Yu Yao (EX-HN 4), He Gu (LI 4), Xia Guan (ST 7), and Jia Cheng Jiang	14 days	1. Reduction of pain intensity 2. Response rate	1. A > B 2. A > B
Zhang (2013) [83]	China	60	1 : 1	52.5	24 : 29	EA + CM	CM (0.3–0.6 g/day dose of carbamazepine)	—	Tou Wei (ST 8) and He Gu (LI 4)	15 days	1. Response rate 2. Adverse events	1. A > B 2. A > B
Wang (2013) [84]	China	40	1 : 1	A: 54.54 ± 2.3 B: 53.92 ± 2.8	A: (3 : 17) B: (5 : 15)	MA + CM	CM (0.2–1.2 g/day dose of carbamazepine)	—	Tai Yang (EX-HN 5)	30 days	1. Reduction of pain intensity 2. Response rate	1. A > B 2. A > B
Luo (2013) [85]	China	40	1 : 1	A: 52.10 ± 12.34 B: 48.30 ± 11.26	A: (7 : 13) B: (6 : 14)	MA	CM (0.3 g/day dose of carbamazepine)	—	Bai Hui (DU 20), Si Shen Cong (EX-HN1), Tai Yang (EX-HN 5), He Gu (LI 4), Wai Guan (SJ 5), Xia Guan (ST 7), Di Cang (ST 4), Si Bai (ST 2), and Cuan Zhu (BL 2)	14 days	1. Reduction of pain intensity 2. Response rate 3. Adverse events	1. A = B 2. A = B 3. A > B
Zhao (2011) [86]	China	60	1 : 1	38–64	27 : 33	MA	CM (0.3 g/day dose of carbamazepine)	—	He Gu (LI 4) and Tai Chong (LR 3)	30 days	1. Reduction of pain intensity	1. A > B
Zheng (2011) [87]	China	24	1 : 1	52.2 ± 17.5	8 : 16	MA	CM (0.6–0.8 g/day dose of carbamazepine)	—	Xia Guan (ST 7)	30 days	1. Response rate	1. A > B
Han (2009) [88]	China	60	1 : 1	—	A: (18 : 12) B: (14 : 16)	EA	CM (0.3 g/day dose of carbamazepine)	—	Xia Guan (ST 7)	10 days	1. Response rate	1. A > B
Zhao (2009) [89]	China	62	1 : 1	—	A: (12 : 19) B: (14 : 17)	MA	CM (0.2–0.4 g/day dose of carbamazepine)	—	Zhong Wan (RN 12) and Guan Yuan (RN 4)	30 days	1. Response rate	1. A > B
Li (2009) [90]	China	50	1 : 1	—	21 : 29	MA + CM	CM (0.6 g/day dose of carbamazepine)	—	Yu Yao (EX-HN 4), Xia Guan (ST 7), Si Bai (ST 2), Di Cang (ST 4), Jia Cheng Jiang, Nei Ting (ST 44), and He Gu (LI 4)	30 days	1. Response rate	1. A > B
Jiao (2008) [91]	China	192	1 : 1	A: 53.38 ± 9.45 B: 51.53 ± 10.83	A: (35 : 61) B: (36 : 60)	MA	CM (0.3 g/day dose of carbamazepine)	—	Quan Liao (SI 18)	30 days	1. Reduction of pain intensity 2. Response rate	1. A < B 2. A < B
Zhang (2006) [92]	China	72	1 : 1	A: 59.3 ± 3.5 B: 57.5 ± 5.8	A: (14 : 22) B: (15 : 21)	MA	CM (0.3 g/day dose of carbamazepine)	—	Quan Liao (SI 18)	24 days	1. Response rate	1. A > B
Zhou (2004) [93]	China	49	31 : 18	A: 42.86 ± 6.28 B: 42.67 ± 5.84	A: (8 : 23) B: (6 : 12)	MA	CM (0.3 g/day dose of carbamazepine)	—	Quan Liao (SI 18)	24 days	1. Response rate	1. A > B

MA: manual acupuncture; EA: electroacupuncture; FA: fire acupuncture; AM: acupuncture-moxibustion; SA: sham acupuncture; CM: conventional medicine.

**Table 2:** Pairwise meta-analysis of reduction of pain intensity.

Comparison		Number	MD (95% CI)	*I* ^2^	*p*

MA	CM	9	**1.14 (0.48, 1.80)** ^*∗*^	89%	<0.00001
MA	AM	1	−0.90 (−1.87, 0.07)	—	—
MA	FA	2	−0.84 (−3.43, 1.75)	86%	0.008
MA	SA	1	**1.66 (0.93, 2.39)** ^*∗*^	—	—
EA	MA	2	0.29 (−1.49, 2.07)	71%	0.06
EA	FA	1	−1.40 (−3.17, 0.37)	—	—
EA	CM	1	**1.07 (0.09, 2.05)** ^*∗*^	—	—
AM	CM	1	−0.08 (−1.19, 1.03)	—	—
MA + EA	MA	1	**3.18 (2.43, 3.93)** ^*∗*^	—	—
MA + EA	EA	2	**1.25 (0.78, 1.72)** ^*∗*^	0%	0.32
MA + CM	CM	7	**1.19 (0.55, 1.84)** ^*∗*^	89%	<0.00001
AM + CM	CM	3	**1.88 (0.87, 2.90)** ^*∗*^	85%	0.001
EA + CM	CM	2	**1.22 (0.42, 2.02)** ^*∗*^	30%	0.23
EA + CM	MA + CM	2	0.61 (−0.62, 1.85)	80%	0.02
MA + CM	SA + CM	1	**1.60 (0.32, 2.88)** ^*∗*^	—	—

^∗^Significant difference. MA: manual acupuncture; EA: electroacupuncture; AM: acupuncture-moxibustion; FA: fire acupuncture; SA: sham acupuncture; CM: conventional medicine.

**Table 3:** Pairwise meta-analysis of response rate.

Comparison		Number	RR (95% CI)	*I* ^2^	*p*
MA	CM	25	**1.21 (1.15, 1.27)** ^*∗*^	44%	0.01
MA	FA	1	0.80 (0.62, 1.02)	—	—
MA	AM	1	0.89 (0.74, 1.08)	—	—
MA	SA	1	**1.68 (1.17, 2.42)** ^*∗*^	—	—
EA	MA	1	1.12 (0.93, 1.35)	—	—
EA	CM	2	1.14 (0.98, 1.52)	0	0.73
AM	CM	1	1.08 (0.84, 1.40)	—	—
AM + CM	CM	3	**1.25 (1.10, 1.42)** ^*∗*^	0%	0.40
MA + CM	CM	10	**1.20 (1.14, 1.26)** ^*∗*^	0%	0.68
EA + CM	CM	4	1.12 (0.92, 1.36)	82%	0.0008
EA + CM	MA + CM	1	1.22 (0.98, 1.52)	—	—
MA + EA	MA	1	**1.50 (1.13, 1.99)** ^*∗*^	—	—
MA + EA	EA	2	**1.33 (1.03, 1.72)** ^*∗*^	0	1

^*∗*^Significant difference. MA: manual acupuncture; EA: electroacupuncture; AM: acupuncture-moxibustion; FA: fire acupuncture; SA: sham acupuncture; CM: conventional medicine.

**Table 4:** The results of network meta-analysis of reduction of pain intensity.

MA + EA										
0.57 (−1.20, 2.35)	FA									
1.37 (−0.47, 3.16)	0.79 (−1.06, 2.58)	AM + CM								
**1.64 (0.48, 2.83)** ^*∗*^	1.07 (−0.46, 2.65)	0.28 (−1.27, 1.91)	EA							
1.75 (−0.03, 3.50)	1.18 (−0.66, 2.95)	0.40 (−1.14, 1.89)	0.11 (−1.45, 1.65)	EA + CM						
**2.25 (0.91, 3.57)** ^*∗*^	**1.68 (0.26, 3.04)** ^*∗*^	0.87 (−0.36, 2.12)	0.60 (−0.48, 1.65)	0.49 (−0.69, 1.69)	MA					
**2.10 (0.54, 3.70)** ^*∗*^	1.54 (−0.13, 3.15)	0.74 (−0.57, 2.07)	0.47 (−0.90, 1.77)	0.35 (−0.64, 1.36)	−0.13 (−1.07, 0.78)	MA + CM				
**2.29 (0.31, 4.20)** ^*∗*^	1.73 (−0.28, 3.71)	0.93 (−0.89, 2.80)	0.66 (−1.13, 2.43)	0.53 (−1.22, 2.35)	0.06 (−1.42, 1.52)	0.19 (−1.42, 1.79)	AM			
**3.68 (0.96, 6.38)** ^*∗*^	**3.11 (0.41, 5.83)** ^*∗*^	2.33 (−0.27, 4.87)	2.03 (−0.57, 4.55)	1.95 (−0.45, 4.32)	1.45 (−0.96, 3.77)	1.59 (−0.60, 3.71)	1.38 (−1.34, 4.07)	SA + CM		
**3.25 (1.84, 4.66)** ^*∗*^	**2.68 (1.16, 4.12)** ^*∗*^	**1.89 (0.79, 2.98)** ^*∗*^	**1.60 (0.45, 2.72)** ^*∗*^	**1.49 (0.45, 2.55)** ^*∗*^	**1.01 (0.40, 1.61)** ^*∗*^	**1.14 (0.46, 1.84)** ^*∗*^	0.96 (−0.51, 2.39)	−0.45 (−2.69, 1.85)	CM	
**3.89 (1.49, 6.27)** ^*∗*^	**3.34 (0.95, 5.76)** ^*∗*^	**2.53 (0.23, 4.92)** ^*∗*^	2.24 (−0.00, 4.49)	2.13 (−0.08, 4.49)	1.64 (−0.28, 3.66)	1.76 (−0.35, 4.00)	1.60 (−0.84, 4.01)	0.20 (−2.86, 3.26)	0.64 (−1.41, 2.77)	SA

^∗^Significant difference. MA: manual acupuncture; EA: electronic acupuncture; AM: acupuncture-moxibustion; FA: fire acupuncture; SA: sham acupuncture; CM: conventional medicine.

**Table 5:** The results of network meta-analysis of response rate.

MA + EA									
2.34 (0.14, 45.48)	FA								
5.27 (0.24, 108.34)	2.31 (0.07, 51.27)	AM							
**3.51 (1.08, 11.91)** ^*∗*^	2.42 (0.15, 33.74)	1.04 (0.09, 15.04)	EA						
**12.43 (2.02, 131.91)**	5.44 (0.78, 57.31)	2.67 (0.43, 20.33)	2.19 (0.47, 19.09)	MA					
11.08 (0.63, 306.10)	5.19 (0.18, 137.33)	2.14 (0.13, 58.84)	1.97 (0.25, 24.41)	0.93 (0.05, 11.76)	MA + CM				
4.57 (0.30, 58.46)	1.98 (0.09, 33.64)	0.85 (0.07, 15.03)	0.79 (0.16, 4.27)	0.37 (0.02, 2.46)	0.41 (0.05, 2.02)	EA + CM			
3.61 (0.23, 47.22)	1.54 (0.06, 26.99)	0.66 (0.05, 11.13)	0.63 (0.11, 3.56)	0.29 (0.02, 1.97)	0.32 (0.03, 2.47)	0.78 (0.17, 3.59)	AM + CM		
**20.24 (1.61, 208.87)** ^*∗*^	8.64 (0.44, 127.99)	3.69 (0.34, 50.71)	3.50 (1.00, 14.47)	**5.03 (3.46, 7.89)** ^*∗*^	**5.10 (2.85, 9.45)** ^*∗*^	**4.32 (1.60, 12.67)** ^*∗*^	**5.53 (1.90, 17.37)** ^*∗*^	CM	
**117.72 (9.06, 2206.40)** ^*∗*^	**49.29 (3.48, 1006.76)** ^*∗*^	**22.23 (1.27, 660.91)** ^*∗*^	**20.21 (2.03, 306.03)** ^*∗*^	**8.89 (1.58, 59.55)** ^*∗*^	10.04 (0.46, 259.77)	**24.94 (1.97, 592.58)** ^*∗*^	**32.11 (2.30, 810.36)** ^*∗*^	5.68 (0.52, 118.12)	SA

^∗^Significant difference. MA: manual acupuncture; EA: electronic acupuncture; AM: acupuncture-moxibustion; FA: fire acupuncture; SA: sham acupuncture; CM: conventional medicine.

**Table 6:** Adverse events in included RCTs.

Interventions	Study (reference)	Sample size	Adverse events
MA	Chen (2021) [38]	40	1 case
	Zhang (2019) [45]	33	1 case of fatigue; 1 case of dizziness; 1 case of drowsiness
	Liu (2019) [51]	44	1 case
	Huang (2018a) [55]	32	1 case of hyperpigmentation
	Yan (2018) [56]	36	1 case of dizziness; 1 case of gastrointestinal reaction; 1 case of fatigue; 1 case of rash; 1 case of pruritus
	Li (2018b) [59]	44	1 case of nausea and vomiting
	Xiao (2016) [67]	50	1 case of dizziness
	Li (2016) [69]	25	1 case
	Zhou (2016) [72]	32	1 case
	Liu (2016) [73]	30	1 case of dizziness; 1 case of fatigue
	Xie (2016) [74]	40	2 cases
	Liu (2015) [75]	42	3 cases of drowsiness
	Wang (2015) [76]	35	2 cases
	Xia (2015) [77]	30	1 case
CM	Chen (2021) [38]	40	6 cases
	Chi (2021) [39]	39	2 cases of drowsiness; 3 cases of dizziness; 3 cases of nausea and vomiting
	Zhang (2019) [45]	29	2 cases of fatigue; 1 case of dizziness; 2 cases of drowsiness; 2 cases of gastrointestinal reaction
	Xu (2019) [48]	33	1 case of dizziness; 1 case of drowsiness; 2 cases of nausea
	Liu (2019) [51]	44	6 cases
	Si (2018) [53]	33	2 cases of peripheral facial paralysis; 4 cases of facial numbness; 5 cases of tinnitus; 6 cases of herpesvirus infection
	Liang (2018) [54]	43	1 case of dizziness; 2 cases of nausea and vomiting; 3 cases of drowsiness; 1 case of fever; 1 case of pruritus
	Huang (2018a) [55]	31	1 case of dermatitis; 5 cases of drowsiness
	Yan (2018) [56]	36	3 cases of dizziness; 4 cases of gastrointestinal reaction; 1 case of fatigue; 2 cases of rash; 3 cases of pruritus
	Li (2018b) [59]	44	1 case of rash; 2 cases of pruritus; 1 case of dizziness; 3 cases of fatigue
	Shen (2016) [66]	40	1 case of dermatitis; 6 cases of dizziness
	Xiao (2016) [67]	50	6 cases of gastrointestinal reaction; 4 cases of abnormal liver function; 2 cases of rash
	Li (2016) [69]	25	5 cases
	Zhou (2016) [72]	32	6 cases
	Liu (2016) [73]	30	1 case of dizziness; 1 case of gastrointestinal reaction; 2 cases of fatigue; 1 case of nausea
	Xie (2016) [74]	40	8 cases
	Liu (2015) [75]	42	3 cases of dizziness; 5 cases of drowsiness; 3 cases of nausea
	Wang (2015) [76]	35	8 cases
	Xia (2015) [77]	30	5 cases
	Zhang (2013) [83]	28	5 cases of dizziness; 6 cases of gastrointestinal reaction
	Luo (2013) [85]	20	3 cases of dizziness; 5 cases of drowsiness; 1 case of xerostomia; 2 cases of nausea and vomiting; 2 cases of anorexia; 2 cases of skin disorders
	Zhao (2009) [89]	20	1 case of dizziness; 3 cases of drowsiness; 3 cases of xerostomia; 4 cases of nausea and vomiting; 3 cases of anorexia; 6 cases of constipation; 1 case of abnormal liver function; 3 cases of abnormal blood; 1 case of abnormal renal function; 2 cases of restless; 8 cases of skin disorders
SA	Liu (2014) [80]	28	1 case of dizziness
	Chi (2021) [39]	39	1 case of dizziness; 1 case of nausea and vomiting
	Shen (2016) [66]	40	2 cases of hyperpigmentation
MA + CM	Zhao (2009) [89]	20	4 cases of dizziness; 7 cases of drowsiness; 6 cases of xerostomia; 1 case of nausea and vomiting; 12 cases of anorexia; 6 cases of constipation; 3 cases of abnormal liver function; 2 cases of abnormal blood; 2 cases of abnormal renal function; 3 cases of restless; 2 cases of skin disorders
EA + CM	Si (2018) [53]	33	1 case of peripheral facial paralysis; 1 case of facial numbness; 3 cases of tinnitus; 2 cases of herpesvirus infection
AM + CM	Xu (2019) [48]	33	2 cases of dizziness

MA: manual acupuncture; EA: electronic acupuncture; SA: sham acupuncture; AM: acupuncture-moxibustion; CM: conventional medicine.

## Data Availability

The data used to support the findings of this systematic review are included within the study.

## Abbreviations

ADDIS: Aggregate Data Drug Information System
AE: Adverse event
AM: Acupuncture-moxibustion
AMED: Allied and Complementary Medicine Database
CBM: Chinese Biomedical Literature Database
CENTRAL: Cochrane Central Register of Controlled Trials
ChiCTR: Clinical Trials and Chinese Clinical Trial Register
CI: Confidence interval
CM: Conventional medicine
CNKI: China National Knowledge Infrastructure
CONSORT: Consolidated Standards of Reporting Trials
EA: Electronic acupuncture
FA: Fire acupuncture
GRADE: Grades of Recommendations, Assessment, Development, and Evaluation
ICC: Intraclass correlation coefficient
MA: Manual acupuncture
MCMC: Markov Chain Monte Carlo
MD: Mean difference
NMA: Network meta-analysis
NRS: Numerical rating scale
PICOS: Participant, intervention, control, outcome, and study design
PRISMA: Preferred Reporting Items for Systematic Reviews and Meta-Analyses
PSRF: Potential scale reduced factor
PTN: Primary trigeminal neuralgia
RCTs: Randomized controlled trials
RevMan: Review Manager
ROB: Risk of bias
RR: Relative risk
SA: Sham acupuncture
SP: Substance P
STRICTA: Standards for Reporting Interventions in Clinical Trials of Acupuncture
VAS: Visual analogue scale
VIP: China Science and Technology Journal Database
WF: Wanfang Database
WHO ICTRP: World Health Organization International Clinical Trials Registry Platform
WOS: Web of Science.

## Supplementary Materials

Supplementary Materials Appendix 1: PRISMA-NMA statement reporting standard checklist. Appendix 2: search strategies of each database. Appendix 3: (a) the PSRF value of reduction in pain intensity and (b) node-splitting test result of reduction in pain intensity. Appendix 4: (a) the PSRF value of response rate and (b) node-splitting test result of response rate. Appendix 5: quality of evidence of reduction in pain intensity in network meta-analysis. Appendix 6: quality of evidence of response rate in network meta-analysis.
